# Deletion of the *c2515* and *c2516* Genes Affects Iron Uptake and Virulence of APEC O1 Strain E516

**DOI:** 10.3389/fvets.2021.654721

**Published:** 2021-04-12

**Authors:** Qingqing Gao, Xi Li, Senyan Su, Lei Yang, Song Gao

**Affiliations:** Animal Infectious Disease Laboratory, Ministry of Agriculture, Jiangsu Co-innovation Center for Prevention and Control of Important Animal Infectious Diseases and Zoonoses, College of Veterinary Medicine, Yangzhou University, Yangzhou, China

**Keywords:** avian pathogenic *Escherichia coli*, iron transport genes, mutant, iron uptake, virulence

## Abstract

Avian pathogenic *Escherichia coli* (APEC), widely spread among poultry, is well-known to cause colibacillosis in chickens, which results in significant losses in poultry industry. The ability to uptake iron in the extra-intestinal environment is prerequisite for APEC survival. For adaptation to the low-iron environments, the bacteria have evolved multiple iron acquisition systems to ensure optimal iron uptake. However, many components of these iron acquisition pathways are still not clearly known. An *in silico* analysis of the genome of a septicemic APEC O1 strain E516 identified two putative iron transport genes homologous to the *c2515* and *c2516* genes from uropathogenic *E. coli* CFT073. In this study, we constructed the single and double gene deletion mutants, and studied their biological characteristic and pathogenic traits through *in vitro* and *in vivo* assays. Reverse transcriptase PCR (RT-PCR) analyses demonstrated that the mutations destroying the reading frame of the target genes abolished their transcription. Deletion of the single or double genes of *c2515* and *c2516* in APEC E516 weakened its ability to produce siderophore. Consistently, the mutants exhibited growth defect under iron-depleted conditions and the intracellular iron levels in the mutants were decreased in comparison with that of the wild-type (WT). Cell infection assays showed that the iron uptake defective mutants were more easily eliminated by the macrophage. Inactivation of the *c2515* and *c2516* genes affected bacterial colonization of chicken tissues, as well as the 50% lethal dose levels compared with the WT strain. Moreover, the expression levels of several iron uptake-related genes were significantly decreased in the double-deletion mutant. In total, the *c2515* and *c2516* may involve in siderophore-mediated iron uptake and participate in the pathogenesis of APEC O1 strain E516.

## Introduction

Avian pathogenic *Escherichia coli* (APEC) causes typical extra-intestinal infections in poultry referred to as colibacillosis, causing severe economic losses and hindering the development of the poultry industry (1). Being the most important bacterial pathogen, APEC is not only affecting variety of bird species, but also threatening the human healthy by sharing multiple virulence factors with human extra-intestinal pathogenic *E. coli* (ExPEC) (2). Among the numerous serogroups identified, the O1, O2, and O78 are the most common serotypes associated with colibacillosis in chickens (3).

Avian pathogenic *E. coli* possesses a variety of virulence genes that produce adhesins, toxins, invasins, serum resistance and iron acquisition factors (4–8). Under infection conditions, the ability of the bacterial pathogen to competitively uptake the endogenous iron determines the outcome of the infections. Iron is an essential nutrient for bacterial growth and plays an important role in regulating numerous cellular processes, including oxygen transport, electron transfer and enzymatic function (9–11). Therefore, effective iron uptake strategy is very critical for the growth and infection of pathogens. Given the limited iron availability, bacteria have evolved a number of iron uptake, transport and utilization systems. Bacteria usually secrete specific high-affinity siderophores to chelate iron and then uptake these iron-binding siderophores into cells *via* specific cellular transport systems (12, 13). The TonB-ExbB-ExbD transport system is required for the energy-dependent transport of ferric siderophores across the outer membrane (14). The SitABCD is a periplasmic binding protein-dependent ABC transport system that mediates iron transport (15). IroN, IreA, and IutA are considered to be outer membrane receptor proteins, which involve in periplasmic internalization of ferri-siderophores (16, 17). Though numerous iron acquisition pathways have been found, the functions and roles of many components are not fully understood.

We previously sequenced the genome of three different serotypes APEC strains: E516 (O1), E058 (O2), and E522 (O78), among which the E516 strain possess a higher virulence than the other two strains. By comparing these genomes, we found that these three strains possess the similar iron acquisition systems, including heme, enterobactin, salmochelin, aerobactin, and yersiniabactin. However, we identified two putative iron transport genes homologous to the *c2515* and *c2516* genes from uropathogenic *E. coli* CFT073, that are present in E516 genome but absent in the E058 and E522 genome. The *c2515*, encoding a ATP-binding cassette (ABC) transporter that mediates iron import, is thought to transport the ferric-siderophores and enter into the cytoplasm with the help of *c2516*, which encoding plasma membrane permeases for high-affinity iron uptake. Whether these two genes contribute to iron uptake and virulence of APEC E516 is unknown. Therefore, in this study, we evaluated the phenotypic characterization of APEC strains containing defined mutations in *c2515* and *c2516*. Our report demonstrate the role they play in iron uptake and virulence of the APEC O1 strain E516.

## Materials and Methods

### Bacterial Strains, Plasmids, and Growth Conditions

All *E. coli* strains, plasmids, oligonucleotide primers, and cell line used in this study are listed in Tables 1, 2. All primers used in amplification of the genes were obtained from Sangon Co. Ltd. (Shanghai, China). *Escherichia coli* was grown on LB agar plates or broth with appropriate antibiotic supplementation. Antibiotics were added at the following concentrations: chloramphenicol, 30 μg/ml and ampicillin, 60 μg/ml.

**Table 1:** Bacterial strains and plasmids used in this study.

**Strain or plasmid**	**Characteristics**	**Source or reference**
**Strains**		
E516	APEC O1 (^a^Ent^+^ Sal^+^ Aer^+^ Ybt^+^)	This study
E516Δ*c2516*	E516Δ*c2516*::*cat*	This study
E516Δ*c2515*	E516Δ*c2515*::*cat*	This study
E516Δ*c2515*Δ*c2516*	E516Δ*c2515*Δ*c2516*::*cat*	This study
ReE516Δ*c2515*Δ*c2516*	complementation of E516Δ*c2515*Δ*c2516*	This study
DH5α	endA1 hsdR17(rk-mk+)supE44 thi-1 recA1 gyrA (NalR) RelA1Δ(lacIZYA-argF) U169deoR (Φ80d lac Δ(lacZ) M15)	Invitrogen
**Plasmids**		
pKD46	Amp; expresses λ Red recombinase	(18)
pKD3	*cat* gene, template plasmid	(18)
pACYC184	Complementary vector, *Cam+*	Gifted
**Cell line**		
HD-11	Chicken macrophage line, chicken myelomonocytic	(19)
	transformed by the myc-encoding MC29 virus	

a *Ent, enterobactin; Sal, salmochelin; Aer, aerobactin; Ybt, yersiniabactin*.

**Table 2:** Primers designed and used in this study.

**Primers**	**Primer sequence 5^**′**^-3^**′**^**	**Target gene, plasmid, or region**
**For deletion**		
VCAT-F	ATGAGCGGGCTTACGATTAATGCGCTGTGCGCCGGTTACGGCAAACGGCttgtgtaggctggagctgct	pKD3
VCAT-R	CTATTTTTCGATTGCACCATCCACTATCACCATCGATCTTCCCTGAGCAatgggaattagccatggtcc	
DCAT-F	TTGGAAAGTACGCTGTCGGTCGAGAACCGCTATCGCCAGCTTTTTCGCCttgtgtaggctggagctgct	pKD3
DCAT-R	TCATGACATACTCCCTCGATGACGCATTACAATGCTCAGAAAGAACGGAatgggaattagccatggtcc	
**For complementation**		
Re*VD**-***184-F	GAGTACTCTATTTTTCGATTGCACCA	*c2516-c2515*
Re*VD**-***184-R	GCCGCGGTGTCGCTCTATCACAGTAT	
**For RT-PCR**		
VF	ATGAGCGGGCTTACGATTAA	*c2515*
VR	CTATTTTTCGATTGCACCAT	
DF	TTGGAAAGTACGCTGTCGGTC	*c2516*
DR	TCATGACATACTCCCTCGATGAC	
**For qRT-PCR**		
Q*chuA*-F	TAGGCCACATCAAGGCTAAAC	*chuA*
Q*chuA-*R	CGGCGACAACTATGTCGTATAA	
Q*fecA*-F	TACTACACCGCCACCAGC	*fecA*
Q*fecA*-R	CTTCTTCGTGCGTGCCTG	
Q*fepC-*F	TCGTTACGCCAGCCATTT	*fepC*
Q*fepC-*R	TGCAGCGCAGACCATAAA	
Q*fyuA*-F	ATGCCTATGTGGGATGGAATG	*fyuA*
Q*fyuA-*R	CCAGTCATCGGTGGTGTATTT	
Q*iroN-*F	GAGACTCTGGTGGTGGAA	*iroN*
Q*iroN-*R	CGAATATCGATCTGGCGG	
Q*irp2-*F	GCGGCTGATTACCAACAATTAC	*irp-2*
Q*irp2-*R	CTGGATCAGGTTGCTCTCTTC	
Q*ireA-*F	GCCATTGAGACTTTCGTCATC	*ireA*
Q*ireA-*R	GATCCAGTCACCGGGTTAAAC	
Q*entA-*F	AGCTGAAACGGAGCGACT	*entA*
Q*entA-*R	TCGGACGCCACAGTGACA	
Q*iutA-*F	AGTATACGCTTTGGGCTCTCA	*iutA*
Q*iutA-*R	TTCGATAACCCAGGTGGTTTG	

*Sca I site and Sac II site are underlined*.

### Construction of Mutant and Complementation Strains

The *c2515* (GI: 1037781) or *c2516* (GI: 1037782) gene was deleted from APEC E516 using gene replacement methods based on the lambda Red recombinase system (18). The chloramphenicol-resistance cassette, flanked by the 5′ and 3′ sequences of the *c2515* or *c2516* gene, was amplified from the pKD3 plasmid using VCAT-F/R and DCAT-F/R primers (Table 2), respectively. The *c2515* or *c2516* single deletion mutant was confirmed by PCR and verified by sequencing.

The *c2515* and *c2516* double-deletion mutant was constructed using the same method as the single mutant strain. The chloramphenicol-resistance cassette, flanked by the 5′ sequences of the *c2516* and 3′ sequences of *c2515* gene, was amplified from the pKD3 plasmid using DCAT-F/VCAT-R primers.

For complementation, the coding sequences of the *c2516-c2515* genes, together with their putative promoter regions, were amplified from strain E516 and cloned independently into pACYC184. The purified recombinant plasmid was transformed into the double-deletion mutant strain to generate the complementation strain.

### RT-PCR

Total RNA was isolated from log-phase bacteria of E516, Δ*c2515*, Δ*c2516*, and Δ*c2515*Δ*c2516*, ReΔ*c2515*Δ*c2516* using an RNeasy kit (Qiagen) and treated with an on-column Rnase-Free Dnase set. The first-strand synthesis of cDNA was primed with random primers using a high capacity cDNA archive kit (Applied Biosystems, Foster City, CA, USA). Primers set for PCR amplification of target genes *c2516, c2515*, and *c2516c2515* in cDNA samples were DF\DR, VF\VR, and DF\VR, respectively (Table 2). In parallel, PCRs were performed with E516 DNA as positive controls and cDNA samples without activation of the reverse transcription as negative controls. The PCR products were resolved on 0.8% agarose gels and visualized by GoodView staining.

### Growth Assay

Growth of all strains in iron-depleted or supplemented medium was examined as previously described (20). Avian pathogenic *E. coli* E516 and its isogenic mutants were cultured overnight in LB broth. Cultures were washed twice and diluted in 10 ml LB, iron-depleted medium [M9 minimum salts, 0.05 mg/ml thiamine, 20% glucose, 0.02 mg/ml L-Tryptopham, 5 mg/ml Casamino acids, 0.1 mM CaCl_2_, 2 mM MgSO_4_ and 200 μM 2,2-dipyridyl (DIP) (Sigma, St. Louis, MO, USA)], iron-depleted medium supplemented with 100 μM FeCl_3_, and the cell density was estimated by spectrophotometry to achieve an approximate starting concentration (OD_600_ = 0.05). Bacterial growth was measured at 1-h intervals over 12 h by spectrophotometry (OD_600_). The experiment was performed in triplicate.

### Siderophore Production

The chrome azurol S (CAS) agar diffusion assay was used for detecting siderophore production (21). CAS agar plate was prepared as follows: first, 60.5 mg CAS [TCI (Shanghai) Development Co., Ltd., Shanghai, China] was dissolved in 50 ml deionized water, and mixed with 10 ml iron III solution (1 mM FeCl_3_, 10 mM HCl). Under stirring, this solution was slowly mixed with 72.9 mg hexadecyltrimetyl ammonium bromide (HDTMA) [TCI (Shanghai) Development Co., Ltd.] dissolved in 40 ml deionized water. The 100 ml resultant dark blue solution was autoclaved, and then 10 ml of that was mixed with an autoclaved mixture of 90 ml water, 1.5 g agarose, 3.024 g Piperazine-1,4-bis (2-ethanesulfonic acid) (PIPES) (Amresco, Solon, OH, USA) and adjusted the pH to 6.8. The CAS agar plate was punched with 5 mm-diameter holes by using a gel puncher. The bacteria of WT strain E516, mutation and complementation strains were cultured to the same OD_600_ of 0.8 in iron-depleted MM9 medium. Culture supernatants of bacteria (50 μl for each) were loaded to wells in the plate, and the plate was incubated at 37°C for 12 h.

### Iron Levels Determination

To determine whether the *c2515* and *c2516* are directly involved in iron uptake, we detected the iron concentrations in the WT, mutant, and complemented strains using an iron colorimetric assay kit (Elabscience Biotech, Wuhan, China). The overnight cultured strains were diluted 1:100 into 100 mL LB media containing 200 μM 2,2′-dipyridyl. Then, the strains were grown to the log phase (OD_600_ = 0.6) and washed three times with PBS. Cell pellets were subsequently obtained and suspended in PBS, and the cells were lysed by ultrasonic crash to release intracellular iron. The samples were centrifugated to exclude the impurities and the supernatant was used for iron concentration determination. Deionized water (0.1 mL), iron standard stock solution (0.1 mL) was diluted according to instructions, and sample (0.1 mL) were individually mixed with a chromogenic agent (0.4 mL), boiled for 5 min, and then centrifuged at 3,000 g for 10 min. The supernatant was subsequently collected. Iron content was estimated by measuring the OD at 520 nm (OD_520_) of the supernatant and a standard product curve (y = ax + b) was created. The following formula was used:

$$\begin{array}{l} \text{Iron concentration }\left(\text{mg}/\text{L}\right)=\left(\text{A}520-\text{b}\right)/\text{a }\times \text{ f}\\ \text{A}520:\text{ sample O}{\text{D}}_{520}-\text{deionized water O}{\text{D}}_{520}\\ \text{f}:\text{ Sample dilution factor} \end{array}$$

### Iron Uptake-Related Gene Expression Analysis

To analyze whether the disruption of *c2515* and *c2516* affects the production of the known iron uptake systems, the iron uptake-related genes (*entA, fepC, irp2, ireA, fecA, fyuA, iutA, chuA, iroN*) were selected for comparing the transcription levels between the WT strain and its isogenic mutants. Total RNA was extracted from the WT, double-deletion mutant and complementation strains cultured in iron-depleted medium. Quantitative real-time PCR (qRT-PCR) was performed to determine the transcription levels of the tested genes using SYBR premix Ex Taq and gene-specific primers (Table 2), and the data were normalized to 16s rRNA. The relative gene expression levels were calculated using the 2^−ΔΔCt^ method.

### Cells Infection Assays

The intracellular survival of the APEC strains was determined using a gentamicin protection assay with chicken macrophage HD-11 cells (19). The cells were cultured in Dulbecco's modified Eagle's medium (Gibco, NY, USA) containing 10% fetal bovine serum (PAA Laboratories, Pasching, Austria), and maintained at 37°C in a 5% CO_2_ environment in 24-well cell culture plates, with 2 × 10^5^ cells per well. The cells were infected with the bacteria at a multiplicity of infection (MOI) of 100 for 1 h to allow the uptake of the bacteria. After the 1 h infection period, the cells were washed three times with PBS, and were incubated with 100 μg/ml gentamicin for 1 h to kill any extracellular bacteria. At this time point (T_0_), the cells were then washed and reincubated with 10 μg/ml gentamicin to prevent extracellular replication, with the incubation proceeding from T_0_ for a further 3, 6, 9, or 12 h. At each time point, the cells were washed with PBS, lysed with 0.1% Triton X-100, diluted in PBS, and plated on LB agar plates for CFU determination. The bacterial internalization ratio was calculated as the percentage of bacteria recovered at T_0_ compared to the bacteria inoculated. Bacterial survival is represented as the survival index, which is the fold-change in bacterial numbers at a specific time point during incubation compared to T_0_.

### Visualization of Surviving Bacteria Within the Macrophage Cells

The HD-11 cells were plated on glass coverslips in 12-well-plates at 2 × 10^5^ cells per well and infected with bacteria at an MOI of 100 CFU per cell as described above. At 12 h post-infection (p.i.), the cells were washed gently with PBS, fixed with 4% para-formaldehyde in PBS, and permeabilized with 0.1% Triton X-100. After blocking with 5% bovine serum albumin, the bacterial cells were incubated with polyclonal chicken anti-APEC O1 serum (1:500) for 1 h at 37°C, washed twice with PBS, and stained with FITC goat anti-chicken IgG (1:500) (Sigma, St. Louis, MO, USA). F-actin was then stained with phalloidin-Alexa Fluor 568 (Thermo Fisher Scientific), and the nuclei were stained with DAPI (4′,6-diamidino-2-phenylindole; Thermo Fisher Scientific). The surviving bacteria in the HD-11 cells were visualized using a laserscanning confocal microscope (Leica TCS SP8 STED).

### LD_50_

For virulence evaluation, the 50% lethal dose (LD_50_) was determined using 1-day-old chick infection model as described previously (22). The chicks were provided with food and water *ad libitum* and treated in accordance with the Regulations for the Administration of Affairs Concerning Experimental Animals. The trial was approved by the Animal Care and Use Committee of Yangzhou University [approval ID: SYXK (Su) 2007–0005, September 21, 2016]. Briefly, the APEC E516 and its isogenic mutants were grown overnight in LB broth, pelleted by centrifugation, and resuspended in PBS to 5 different densities of 10^8^, 10^7^, 10^6^, 10^5^, 10^4^ CFU/mL, respectively. For each bacterial density, 6 1-day-old chicks were inoculated into the air sacs with 0.1 mL of each bacterial suspensions, respectively. Bacterial CFUs in the injected inoculum were confirmed by plating on LB agar. Mortality was monitored until 7 days post-infection. LD_50_ results were calculated using the method by Reed and Muench (23).

### The Colonization Ability of the Mutants

We further evaluated the role of these genes in APEC colonization and virulence. Briefly, 3-week-old white leghorn SPF chickens were inoculated into the left thoracic air sac with suspension containing 10^7^ CFU of the WT strain E516 or mutant derivatives. After 24 h of infection, 10 chickens of each group were euthanatized and hearts, livers, spleens, lungs, and kidneys were aseptically collected. Samples were weighed, triturated and homogenized. The number of the bacteria colonized in the organs was determined by plating 10-fold serial dilutions of the homogenates on LB agar plates.

### Statistical Analysis

Differences between groups were analyzed using the commercially available statistical software GraphPad Prism v7.0 (GraphPad Software). *P* < 0.05 was considered statistically significant and *P* < 0.01 was highly significant.

## Results

### Genetic Analysis and Construction of the Single- and Double-Deletion Mutants for *c2515* and *c2516*

**Figure 1:**
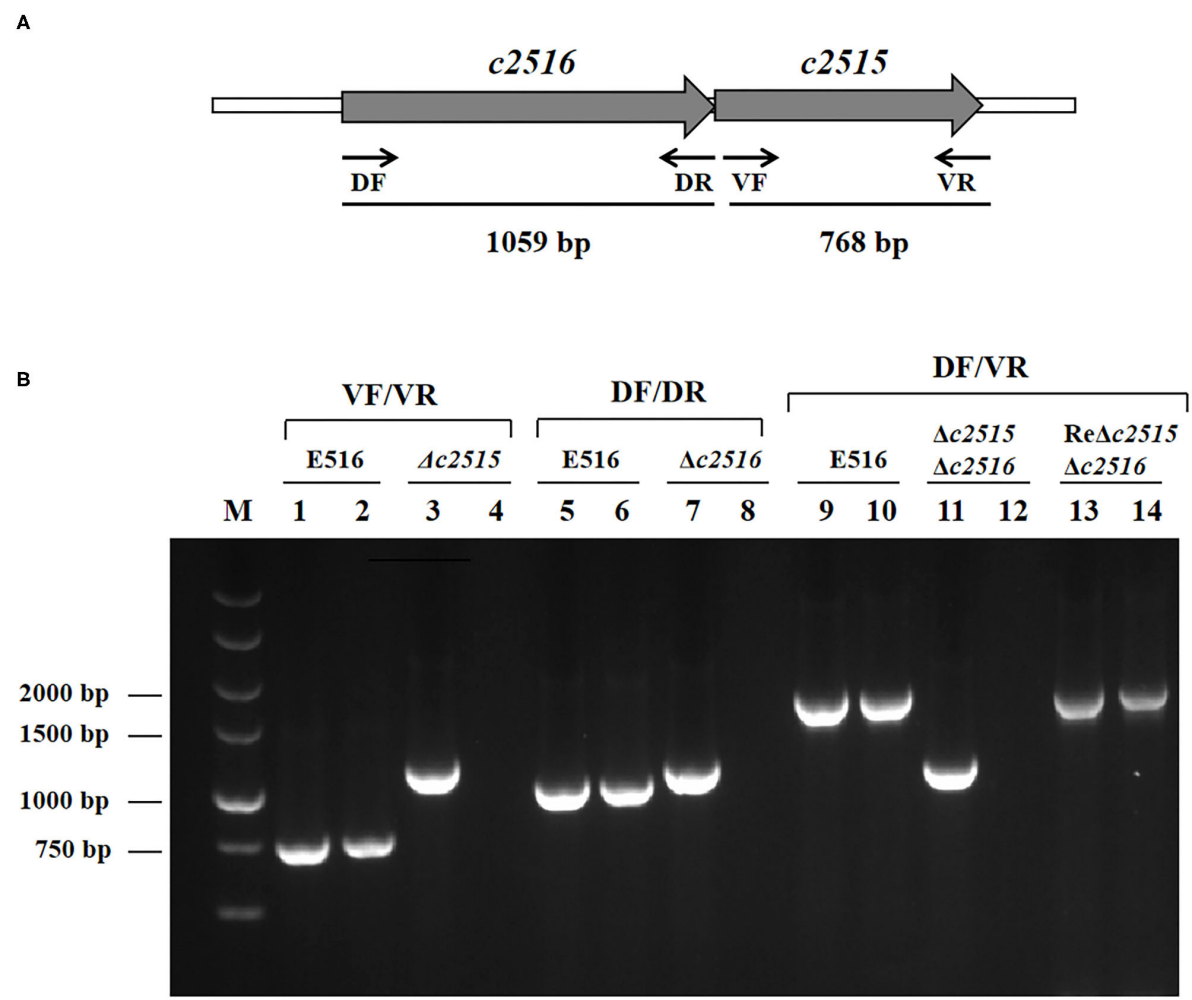
**(A)** Genetic organization of the *c2515* and *c2516* loci in the APEC E516. Large arrows represent the direction of transcription, and the sites of primers used for detection are indicated with small arrows. **(B)** Detection of target genes transcription by RT-PCR. Templates: lanes 1, 5, 9: genomic DNA from E516; lanes 2, 6, 10: cDNA derived from total RNA of E516. Lanes 3, 7, 11, 13: genomic DNA from Δ*c2515*, Δ*c2516*, Δ*c2515*Δ*c2516*, ReΔ*c2515*Δ*c2516*, respectively. Lanes 4, 8, 12, 14: cDNA derived from total RNA of Δ*c2515*, Δ*c2516*, Δ*c2515*Δ*c2516*, ReΔ*c2515*Δ*c2516*, respectively. Target genes: Lanes1–4: *c2515*; Lanes 5–8: *c2516*; Lanes 9–14: *c2516c2515*.

Based on the sequenced genome of APEC O1 strain 516, primers DF/DR and VF/VR were designed to amplify two putative iron transport genes 0199 and 0200. PCR detection showed that these two genes present in the genome of E516, but absent in the genome of E058 and E522 (Data not shown). The nucleotide homology was assessed by searching the non-redundant nucleotide collection at GenBank. The searches revealed that the 0199 and 0200 genes share 100% identity with the uropathogenic *E. coli* CFT073 *c2515* and *c2516* genes, respectively. These two genes are adjacently located, with the *c2516* being the upstream gene of *c2515* (Figure 1A).

After construction of the mutants by λ Red recombinase-mediated mutagenesis, sequencing analysis confirmed that the Cat cassette had been inserted into the genome at the predicted position. RT-PCR analysis demonstrated that the insertion had disrupted the expression of the target genes (Figure 1B). These mutants were named as E516Δ*c2515*, E516Δ*c2516*, and E516Δ*c2515*Δ*c2516*, respectively.

### Growth in Iron-Depleted and Iron-Supplemented Medium

**Figure 2:**
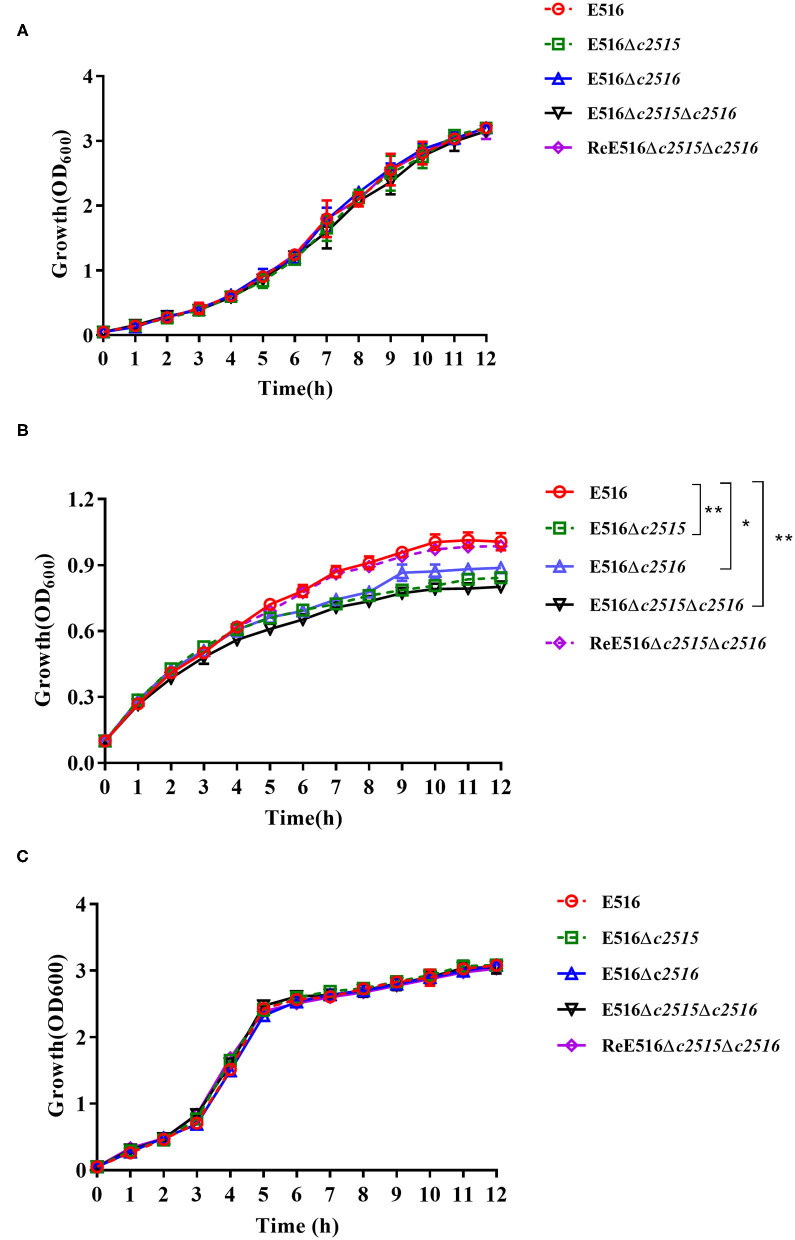
Growth curves of E516 wild-type strain and its mutants. **(A)** The E516, Δ*c2515*, Δ*c2516*, Δ*c2515*Δ*c2516*, and ReΔ*c2515*Δ*c2516* strains were grown in LB broth at 37°C, and their growth was determined by measurement of optical density at 600 nm (OD_600_). **(B)** Growth curves of the E516 and its mutants in iron-depleted MM9 medium. **(C)** Growth curves of the E516 and its mutants in iron-depleted MM9 medium supplemented with 100 μM FeCl_3_. The data represent averages of three independent assays (*P < 0.05, **P < 0.01).

To determine whether these two genes play roles in iron uptake, the growth of strains with either a single deletion of *c2515* or *c2516*, or the double deletion of both genes, was compared. Growth of all strains in iron-depleted and iron-supplemented medium was observed by measurement of OD_600_ every hour for 12 h of cultivation. The results showed that all three mutants grew at rates similar to that of their wild-type strain in LB medium (Figure 2A). However, they manifested significantly retarded growth compared with the WT strain under the iron-depleted culture conditions, while the double-mutant grew more slowly than either single-mutant strain (Figure 2B). The mutant strains' growth defects in iron-depleted medium were reversed by supplementation with 100 μM FeCl_3_ (Figure 2C).

### The Siderophore Production and Cellular Iron Uptake

**Figure 3:**
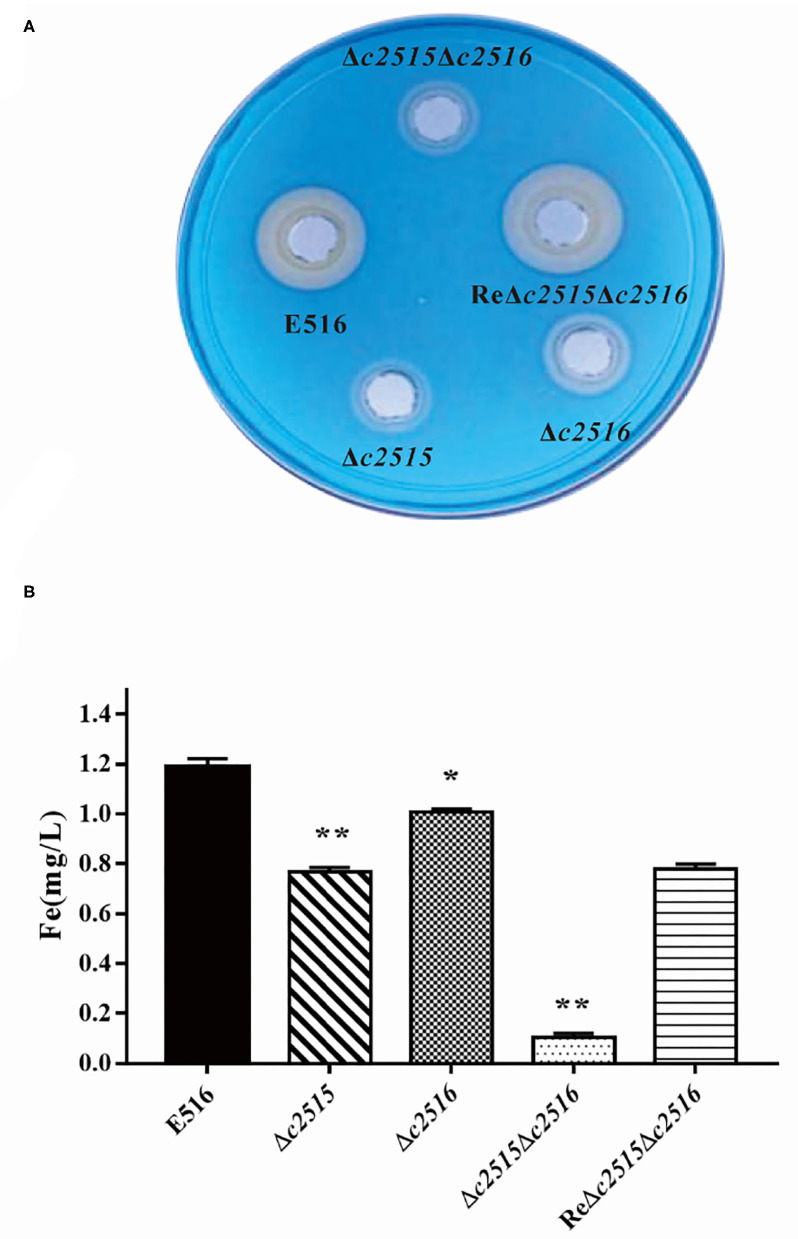
The iron uptake ability between the WT and the mutants strains. **(A)** The CAS agar diffusion assay. Strains E516 and ReΔ*c2515*Δ*c2516* complementation rendered orange halos larger than that of the mutants. **(B)** The intracellular iron levels of the WT, mutant, and complemented strains. The bacterial strains were cultured in iron-depleted MM9 medium to the log phase. The cellular iron contents of the WT, mutant, and complemented strains were determined using an iron colorimetric assay kit (*P < 0.05, **P < 0.01).

The CAS agar diffusion assay was adopted to detect the siderophore production. The orange halo produced by the bacterial strains on CAS agar is indicative for the siderophore production. After culture, the orange halos of the WT strain E516 and the complementation strain were significantly larger than those of the mutant strains on the CAS plate (Figure 3A), indicating that the *c2515* and *c2516* genes may involved in the production of the siderophore.

We tested further, whether the deletion of these two genes would affect the cellular iron uptake. Our results demonstrated that, when mutant cells grew in LB-Fe medium (LB+DIP), they had about 2–7-fold less free intracellular iron than WT cells. Furthermore, the free intracellular iron level is dramatically decreased in the Δ*c2515*Δ*c2516* mutant as compared with either the single-mutant strain under iron limitation (Figure 3B). Iron uptake by the complemented strain was partially restored. Thus, these two genes displayed a synergistic effect on iron uptake in APEC E516.

### Transcription Levels of Iron Uptake-Related Genes in the Mutant

**Figure 4:**
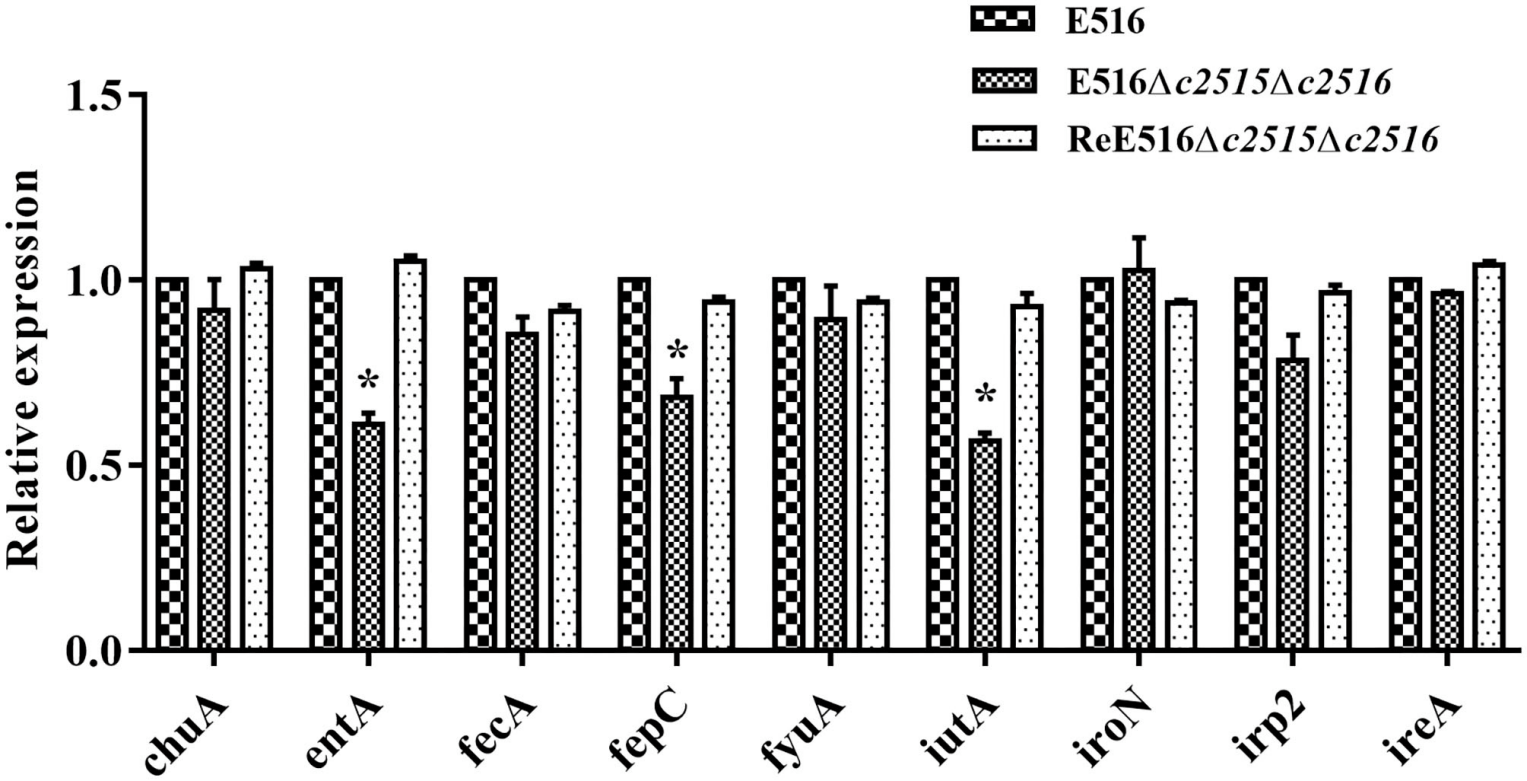
Quantitative real-time reverse transcription-PCR (qRT-PCR) analysis of gene expression. The expression levels of the iron uptake-related genes (*entA, fepC, irp-2, ireA, fecA, fyuA, iutA, chuA, iroN*) in the WT strain, Δ*c2515*Δ*c2516*, and ReΔ*c2515*Δ*c2516* were tested by qRT-PCR. The relative gene expression levels were calculated using the 2^−ΔΔCt^ method (*P < 0.05).

The effects of disruption of *c2515* and *c2516* on the expression of the iron uptake-related genes were further tested under iron-depleted conditions using qRT-PCR. Our data showed that the expression levels of *entA, fepC*, and *iutA* were significantly decreased in the double mutant by 0.61, 0.68, and 0.56 times, respectively (*P* < 0.05), while those of the other iron uptake-associated genes were not significantly changed compared to the wild-type strain. The expression levels of these genes were restored in the complementation strain (Figure 4).

### Iron Uptake-Defective Mutants Survive Poorly Within Chicken Macrophages

**Figure 5:**
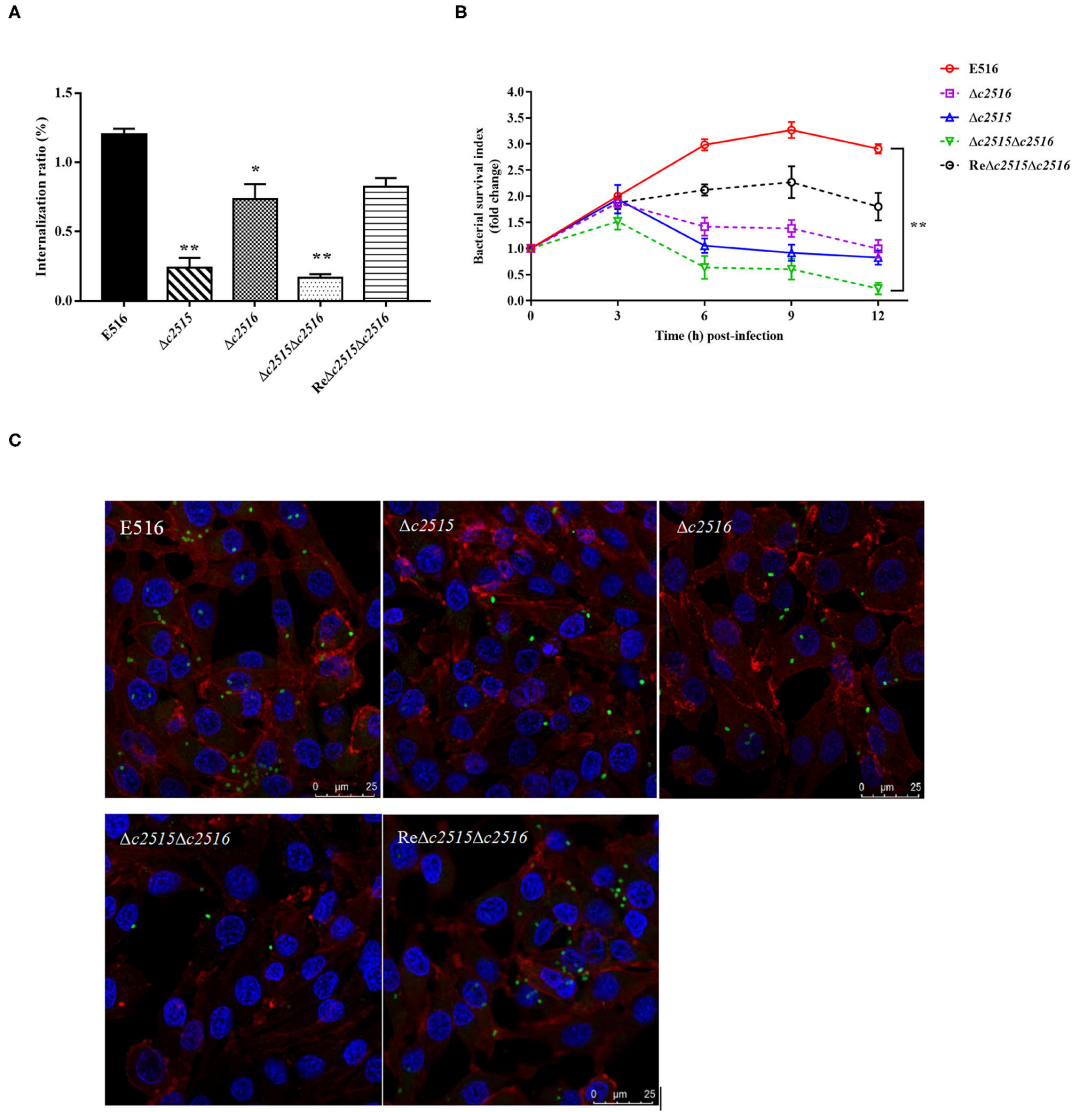
Intracellular survival of bacteria in HD-11 chicken macrophages. **(A)** Bacterial internalization into HD-11 cells. **(B)** The intracellular survival of the WT strain E516, the isogenic Δ*c2515*, Δ*c2516*, and Δ*c2515*Δ*c2516* mutants, and the complemented strain ReΔ*c2515*Δ*c2516*, respectively, were compared over a 12 h period. The standard errors of the means of three independent experiments are shown. Asterisks indicate statistically signifificant differences (**P < 0.01, *P < 0.05). **(C)** Laser-scanning confocal microscopy visualization of the surviving bacteria in the HD-11 macrophage cells at 12 h post-infection. Bacteria were incubated with polyclonal chicken anti-APEC O1 serum and stained with FITC goat anti-chicken IgG, after which the infected cells were treated with DAPI (nuclei staining) and phalloidin-Alexa Fluor 568 (actin staining).

To understand the effect of iron uptake on the intracellular survival of APEC, we compared the bacterial yields of mutants and the WT strains within the chicken macrophages. Initially, the Δ*c2515* or Δ*c2516* single and Δ*c2515*Δ*c2516* double mutants showed significantly decreased internalization compared with the WT strain (Figure 5A). During the following survival period, both the single and double mutants survived poorly within the HD-11 macrophages, as showed by the lower bacterial yield recovered at each specified time point (Figure 5B). The intracellular survival of the complementation strain was higher than that of the single or double mutants strains, but it did not return completely to the wild-type level. These results were further confirmed by immunofluorescence analysis in which HD-11 macrophages displayed higher intracellular bacterial loads of WT or complemented strain than the resulting single or double mutants at 12 h post-infection (Figure 5C). These results showed that knockout of the *c2515* or *c2516* genes impaired the intracellular survival of APEC E516 in macrophages.

### Evaluation of the Virulence of Mutants by LD_50_

According to the LD_50_ results, the WT strain E516 exhibited a high lethality in birds, while the knockout mutants of these two genes reduced the virulence of E516 in varying degrees. A 7-fold reduction of the virulence was observed in the Δ*c2515* mutant, whereas the reduction was 5-fold in absence of *c2516*. The double mutant showed a more dramatic decrease in virulence by 10-fold (Table 3), while the complementation of the double mutant restored its virulence.

**Table 3:** LD_50_ of wild type strain APEC E516 and its isogenic mutants.

**Strain**	**Challenge dose (CFU)**					**LD_50_**
	**10^7^**	**10^6^**	**10^5^**	**10^4^**	**10^3^**	
APEC E516	6/6^*^	6/6	6/6	5/6	3/6	10^3.045^
E516Δ*c2516*	6/6	6/6	6/6	3/6	2/6	10^3.535^
E516Δ*c2515*	6/6	6/6	5/6	3/6	1/6	10^3.880^
E516Δ*c2515*Δ*c2516*	6/6	6/6	5/6	2/6	1/6	10^4.029^
ReE516Δ*c2515*Δ*c2516*	6/6	6/6	6/6	3/6	3/6	10^3.425^

* *Number of dead chicks/Number of inoculated chicks*.

### The Colonization Ability of the Mutants

**Figure 6:**
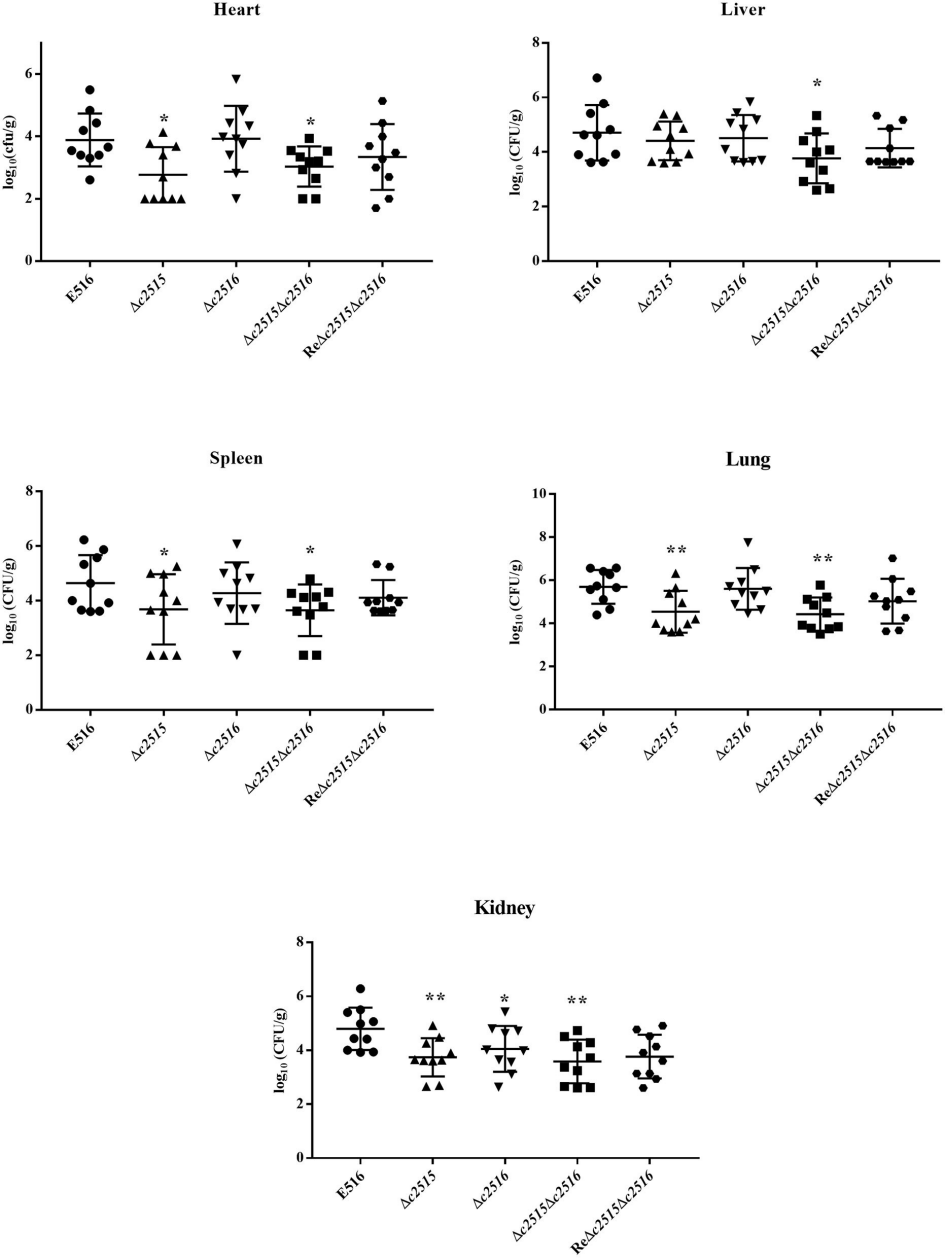
Colonization of the WT strain E516 (•), Δ*c2515* (▴), Δ*c2516* (▾), Δ*c2515*Δ*c2516* (■) and ReΔ*c2515*Δ*c2516* (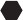) during systemic infection. A, Heart; B, Liver; C, Spleen; D, Lung; E, Kidney. Data were presented as log_10_ (CFU/g) of tissues. Horizontal bars indicated the mean values. Each data represented a sample from an individual chicken. Statistically significances as determined by the Mann-Whitney-test were indicated by asterisks (*P < 0.05; **P < 0.01).

The colonization ability was assessed in WT strain and mutants of *c2515* and *c2516* using the chicken septicemia model. At 24 h post-infection, the WT strain E516 colonized more efficiently in the internal tissues, with the bacterial loads in the selected organs ranged from 7.7 × 10^5^ to 1.1 × 10^4^ CFU per gram of tissues. Compared with the WT strain E516, the mutant E516Δ*c2515*Δ*c2516* showed significantly reduced bacterial numbers in all tested organs, with the bacterial loads ranged from 1.5 × 10^4^ to 4.6 × 10^2^ CFU per gram of tissues (*P* < 0.05, Figure 6). Bacterial loads of Δ*c2515* in the heart (2.4 × 10^2^ CFU.g-1), spleen (4.1 × 10^3^ CFU.g-1), lung (1.6 × 10^4^ CFU.g-1) and kidney (7.9 × 10^3^ CFU.g-1) were significantly lower than those in WT colonized organs (*P* < 0.05, Figure 6). The Δ*c2516* mutant showed a decreased colonization in kidney of challenged chickens than the WT bacteria (*P* < 0.05, Figure 6). The complementation strain ReΔ*c2515*Δ*c2516* colonized the chicken tissues similarly to the WT strain (*P* > 0.05, Figure 6).

## Discussions

Avian pathogenic *E. coli* utilize a variety of virulence factors to enhance its infection. However, strains of different serogroups or genotypes may possess different virulence potentials. In our previous study, we have identified the O1 strain E516 that are of substantially greater virulence than O2 strain E058 and O78 strain E522. We further explored the genomic difference that might underlie this difference in virulence. By comparing the genomes of these three strains, we identified two putative iron transport genes *c2515* and *c2516* that are located on the genomes of E516, but absent in the genomes of E058 and E522. Sequence blast in NCBI database showed that these two genes were also found in several other ExPECs such as uropathogenic *E. coli* UTI89 and the neonatal meningitis *E. coli* O18. Because iron plays significant roles in metabolism, the ability of APEC to sequester iron from their hosts is a major virulence determinant (24). In this study, we sought to evaluate the role of these two genes in iron uptake and virulence of APEC E516.

The iron chelator dipyridyl was used to produce iron-limited conditions that inhibited the growth of APEC strains. Our data suggest that *c2515* or *c2516* is necessary for efficient growth of APEC E516 under iron limitation but not under iron sufficiency. Since the chelator may also sequester other metals, addition of extra-iron to the iron-depleted medium for a regain of growth is a more direct way to confirm that the drop in growth is likely to be due to a decrease in ability to sequester iron. Indeed, the optical density of cultures of the mutant strains in iron-depleted medium supplemented with FeCl_3_ regained levels similar to that of the wild-type strain, suggesting that the mutants strain's growth defect in MM9 medium where dipyridyl was added was the results of iron depletion. Because of the observed growth defect under iron-limited conditions, we analyzed siderophore production in mutant strains lacking one or two genes. Phenotypical analysis revealed a weak CAS halo in the either single- or double-deletion strains, while complementation restored the ability of the double mutant to produce a CAS halo similar to that of WT strain. A defect in siderophore production might result in lowered iron transport and enrichment in the bacterial cells. Indeed, the levels of iron concentration observed in the isogenic deficiency mutants were significantly lower when compared to the WT strain containing the functional endogenous iron transport systems. These results showed *c2515* and *c2516* conferred a significant increase in uptake of ferric under iron-limited conditions, suggesting they may function as a ferric iron transporter. Thus, *c2515*, in combination with *c2516*, is critical for growth under low-iron conditions and this effect may be mediated by favoring siderophore production in APEC.

As bacterial cells require iron for basic cellular processes such as respiration, defects in cellular iron uptake and utilization would decrease bacterial viability (25). In this study, the chicken-derived macrophages were infected with the WT and its derivative mutants strains. At the indicated times following infection, the macrophages were lysed, and the bacterial viability was determined. The results showed that the bacterial proliferation in macrophages were impaired by mutation of the *c2515* and/or *c2516* genes, indicating that iron uptake is involved in stimulation of bacterial viability.

Although the pathogenic mechanism of APEC is complex, increasing evidence has demonstrated that iron acquisition systems are important for APEC infectivity (15, 16). The well-established chicken infection model were used to assess the relative and combined contributions of these genes to APEC pathogenesis. In the 1-day-old chicks lethality model, the double mutants exhibited lowered virulence than any single mutant, which are all less virulent than the WT strain. We next examined the ability of the mutants to colonize 3-week-old chickens. The *c2515* and/or *c2516* mutants showed significantly decreased colonization compared with the wild-type strain in various organs tested in a single-strain challenge model. The complementation strain restored the virulence, but it did not fully reach the levels of WT strain. It is possible that the reduced level of regain of the phenotypes may be due to poor or different expression of these genes in the complemented strains. As well, the complementation plasmid may be instability in the absence of selective antibiotic pressure. Without antibiotic pressure, the plasmid could be lost, resulting in a reduced level of complementation due to loss of the genes from a portion of the cell population. These results show that disruption of *c2515* and *c2516* may attenuate APEC virulence in chickens, indicating that they are required for systemic infection in the chicken infection model.

As the product of *c2515* and *c2516* are putative iron transporters, which are known to mainly partake in uptake ferric-siderophores from the periplasm into the cytoplasm. It is unclear how such genes could affect production of siderophores. The E516 strain produce four types of siderophores, including enterobactin, yersiniabactin, salmochelin and aerobactin, and use exogenous siderophores as well as citrate. It is important to know how or why loss of the *c2515* and *c2516* genes can contribute to decreased growth in iron-depleted medium and reduced virulence considering numerous iron systems are present in APEC E516. Through qRT-PCR analysis, we found that knockout of these genes somehow significantly reduced the expression of *entA, fepC*, and *iutA*, which implied that the enterobactin and aerobactin may be compromised or reduced in the double mutant. However, the concrete mechanism for these affections is unclear and need further study.

Taken together, our results indicate that *c2515* and *c2516* may involve in iron uptake and contributes to APEC virulence, suggesting the potential of these systems to act as targets for novel antimicrobial agents.

## Data Availability Statement

The original contributions generated in the study are included in the article/supplementary material, further inquiries can be directed to the corresponding author.

## Ethics Statement

The animal study was reviewed and approved by Animal Care and Use Committee of Yangzhou University [Approval ID: SYXK (Su) 2007–0005, September 21, 2016].

## Author Contributions

QG designed the experiments and drafted the manuscript. XL carried out the main experiments. SS performed the statistical analysis. LY participated in the chicken infection assays. SG reviewed the manuscript and gave instructions in this study. All authors contributed to the article and approved the final manuscript.

## Conflict of Interest

The authors declare that the research was conducted in the absence of any commercial or financial relationships that could be construed as a potential conflict of interest.
